# Microwave resonant absorption of SARS-CoV-2 viruses

**DOI:** 10.1038/s41598-022-16845-5

**Published:** 2022-07-22

**Authors:** Peng-Jui Wang, Yu-Hao Pang, Sheng-Yu Huang, Jun-Tung Fang, Sui-Yuan Chang, Shin-Ru Shih, Tian-Wei Huang, Yi-Jan Chen, Chi-Kuang Sun

**Affiliations:** 1 Graduate Institute of Photonics and Optoelectronics and Department of Electrical Engineering, National Taiwan University, Taipei, 10617 Taiwan; 2 Department of Clinical Laboratory Sciences and Medical Biotechnology, National Taiwan University College of Medicine, Taipei, 100 Taiwan; 3 Research Center for Emerging Viral Infections, College of Medicine, Chang Gung University, Taoyuan, 33302 Taiwan; 4 Department of Medical Biotechnology and Laboratory Science, College of Medicine, Chang Gung University, Taoyuan, 33302 Taiwan; 5 Department of Laboratory Medicine, Linkou Chang Gung Memorial Hospital, Taoyuan, 333 Taiwan; 6 Research Center for Chinese Herbal Medicine, Chang Gung University of Science and Technology, Taoyuan, 33303 Taiwan; 7 Research Center for Food and Cosmetic Safety, Chang Gung University of Science and Technology, Taoyuan, 33303 Taiwan; 8 Graduate Institute of Health Industry Technology, College of Human Ecology, Chang Gung University of Science and Technology, Taoyuan, 33303 Taiwan; 9 Department of Laboratory Medicine, National Taiwan University Hospital, Taipei, 100 Taiwan; 10 Graduate Institute of Communication Engineering and Department of Electrical Engineering, National Taiwan University, Taipei, 10617 Taiwan; 11 Molecular Imaging Center and Graduate Institute of Biomedical Electronics and Bioinformatics, National Taiwan University, Taipei, 10617 Taiwan

**Keywords:** Biological techniques, Biophysics, Microbiology, Health care

## Abstract

Low power microwave can effectively deactivate influenza type A virus through the nonthermal structure-resonant energy transfer effect, at a frequency matching the confined-acoustic dipolar mode frequency of the virus. Currently, aerosol is considered the major route for SARS-CoV-2 transmission. For the potential microwave-based sterilization, the microwave-resonant frequency of SARS-CoV-2 must be unraveled. Here we report a microwave absorption spectroscopy study of the SARS-CoV-2 and HCoV-229E viruses through devising a coplanar-waveguide-based sensor. Noticeable microwave absorption can be observed, while we identified the resonant frequencies of the 1st and 2nd dipolar modes of SARS-CoV-2 virus as 4 and 7.5 GHz respectively. We further found that the resonant frequencies are invariant to the virus titer, and we also studied the microwave absorption of HCoV-229E in weak acidity medium to simulate the common pH value in fluid secretion. Our results suggest the possible radiation frequency for the recently proposed microwave sterilization devices to inactivate SARS-CoV-2 virus through a nonthermal mechanism so as to control the disease transmission in the post-pandemic era.

## Introduction

Widely considered the major route, airborne transmission of SARS-CoV-2 is made possible because aerosol particles are small enough to float in air^1^. Under the current circumstances that the new variant of concern, such as Omicron (B.1.1.529)^2^ variant, may be vaccines resistant, controlling the pandemic once again requires basic actions such as maintaining a physical distance, wearing appropriate masks, regular hand washing, and sterilization^3^. Generally, chemical and physical sterilization processes are limited by the spatial coverage and penetration, and they also cause harm to humans and animals. Therefore, in order to lessen the risk of getting infected in various scenarios without facial mask in the post-pandemic era, an uninterrupted and safe sterilization method with an excellent penetration capability to sterilize free spaces is urgently needed.

Interactions of electromagnetic waves with materials typically involve energy transfer. Previous studies demonstrated that microwaves can cause viruses with simple geometries to resonate through the structure-resonant energy transfer (SRET) effect, at a frequency matching its confined-acoustic dipolar mode frequency^4–9^. A recent study further revealed that 8.4 GHz microwaves with power density 810 W/m^2^ can efficiently rupture the influenza A virus membrane through the SRET effect, thereby achieving at least 3-log reduction to the active virus in less than 15 minutes^6^. SARS-CoV-2 virions have been reported to remain stable and infectious in aerosol for up to 3 hours^10^; therefore, microwaves with specific frequencies could potentially be used to inactivate SARS-CoV-2 under reasonable microwave power densities that are safe for the human body. Various devices and methodologies have been designed and proposed in order to inspect the inactivation efficacy of the SARS-CoV-2^11–14^, but the correct resonant frequencies remained unknown. Considering the microwave sterilization in public area, microwave frequencies that would resonate with the biological systems should be avoided and the microwave exposure should follow the IEEE safety standards. It is thus highly desirable that the resonant frequencies of SARS-CoV-2 could be determined by quantitatively assessing the microwave resonant absorption (MRA) spectra.

In this report, significant microwave absorption by the SARS-CoV-2 virus was observed at corresponding microwave resonant frequencies. We identified two resonant frequencies, 4 and 7.5 GHz, on the normalized insertion loss (IL) spectra with a magnitude up to 32% of the fundamental resonant frequency, which is commensurate with that previously reported for higher densities Influenza A H3N2 virus^6^. Our results revealed a highly coupled mechanism, wherein energy from the microwave electric field causes SARS-CoV-2 viral structural resonance; while the bandwidth of the MRA frequency also coincided with the size inhomogeneity of the virus. We also investigated the effects of virus titer and the medium acidity using human corona virus 229E (HCoV-229E).

## Results/discussion

### The 1st and 2nd order dipolar modes frequencies of SARS-CoV-2 virion are identified by the normalized insertion loss spectra

**Figure 1:**
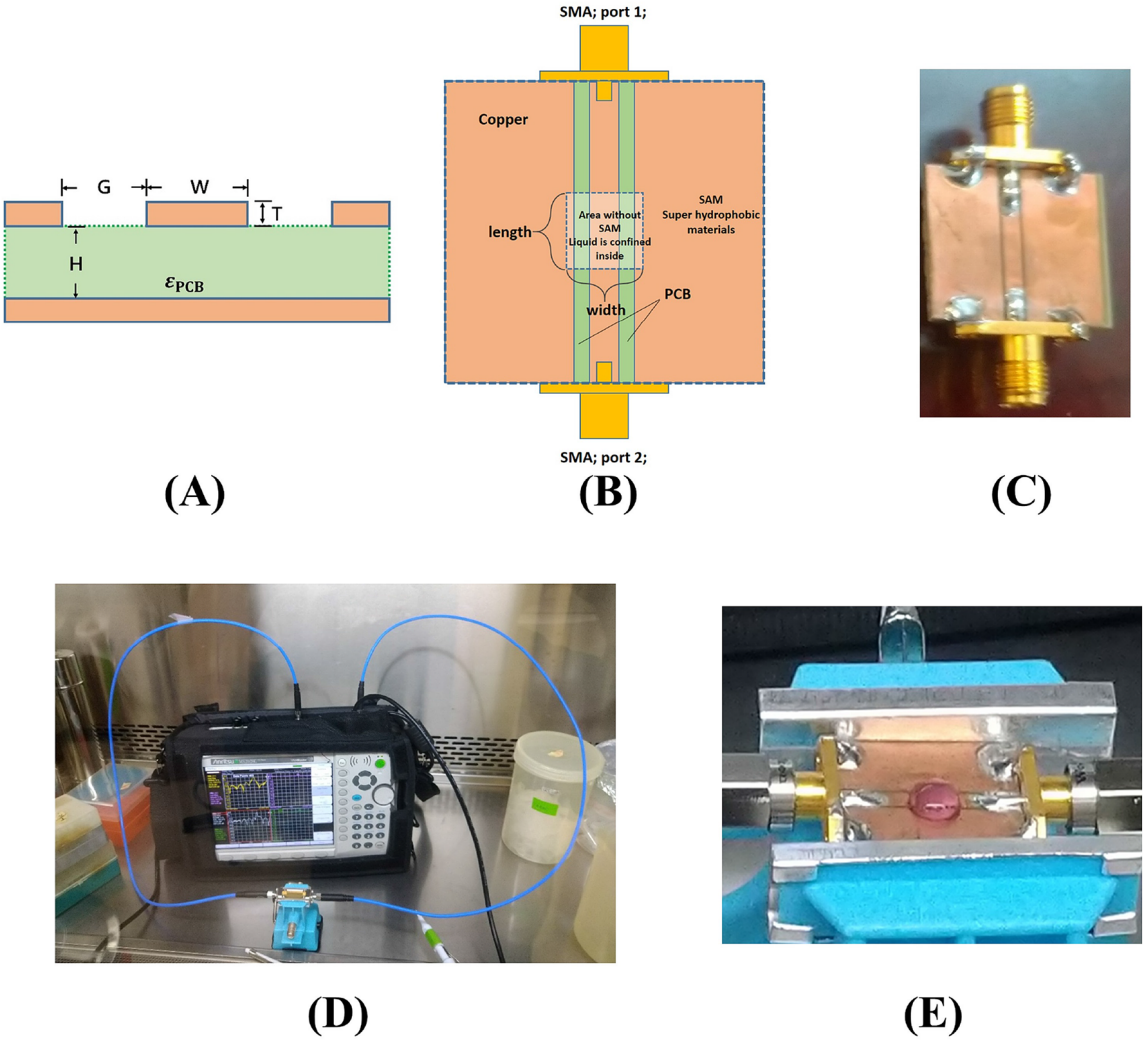
(**A**) Schematic showing the cross-section of the coplanar waveguide. (**B**) Schematic showing the design of the CPW with self-assembled monolayer mask on top to expose the gap and the signal line. (**C**) CPW with hydrophobic SAM mask before connecting to the VNA. (**D**) Microwave absorption measurement system set up in a biological safety cabinet in BSL-2 laboratory. (**E**) 40 µL DMEM medium droplet with coronavirus confined inside the SAM mask.

**Figure 2:**
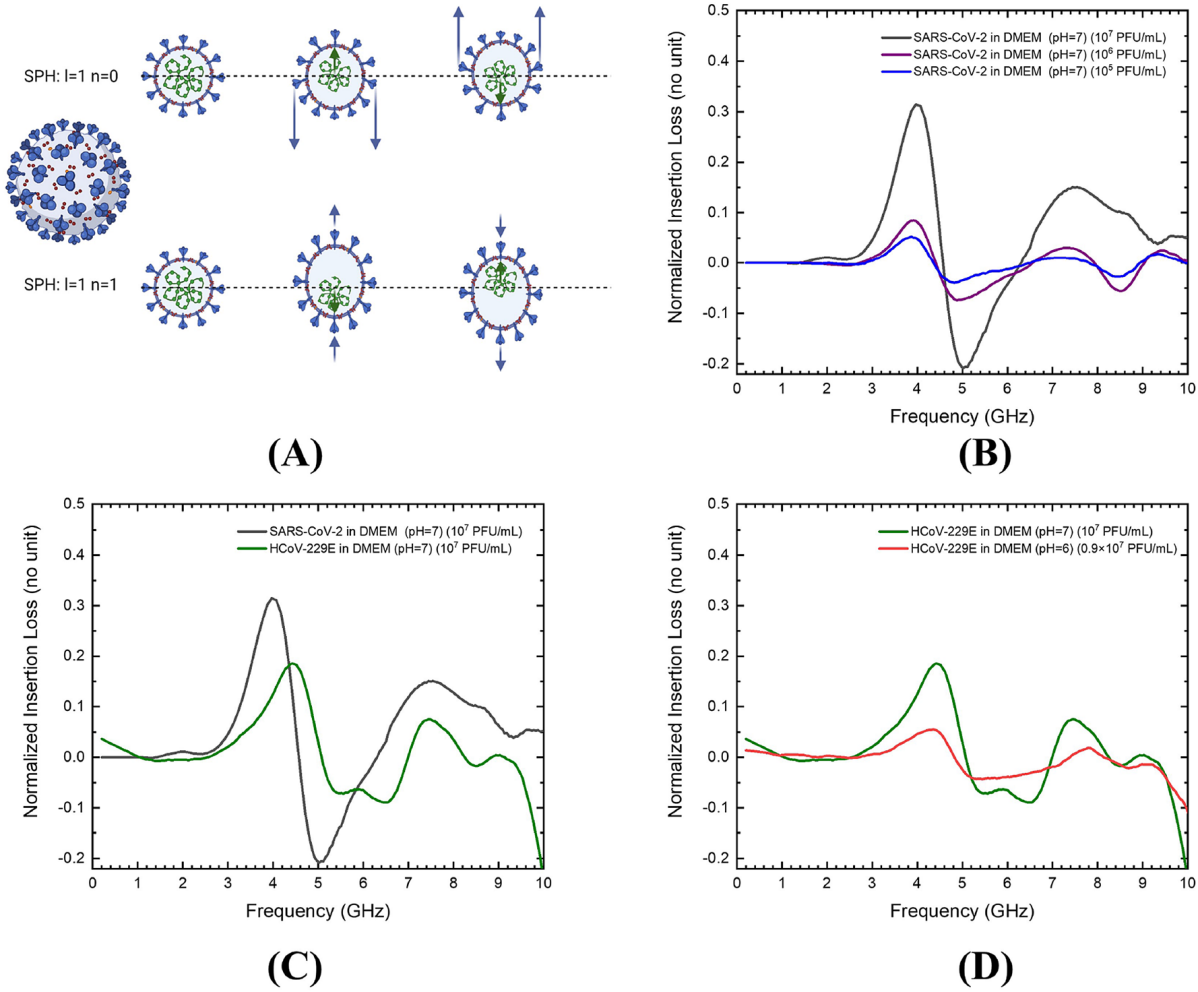
The resonance process of SARS-CoV-2 and its microwave absorption spectra for different concentrations compared with HCoV-229E under different experimental conditions. (**A**) Schematic illustrating the displacement of two dipolar modes of the spherical particle, with angular momentum *l* = 1 and with quantum numbers n = 0 or 1, driven by the electric field generated by the microwave. The blue and green arrows represent the displacements of the shells and the core respectively. Under the viewpoint of two-body problem, for the vibration mode of n = 0, the motion of the virion is like an inner cylinder and an outer ring that are moving in opposite directions. For the other vibration mode of n = 1, the motion behaves like a core and a shell that are moving in opposite directions. (**B**) Normalized insertion loss for different concentrations of SARS-CoV-2 (10^7^ PFU/mL, black-colored line; 10^6^ PFU/mL, purple-colored line; and 10^5^ PFU/mL, blue-colored line) purified in DMEM. Every spectrum in the figure represents one measurement of the normalized IL. (**C**) The normalized insertion loss of SARS-CoV-2 (black-colored line) and HCoV-229E (green-colored line) with the same viral concentration (10^7^ PFU/mL) and purified in the same medium DMEM (pH 7.4). The dataset of SARS-CoV-2 in this plot is the same as (**B**) for the comparison. (**D**) The normalized insertion loss of HCoV-229E in DMEM with different acidity levels. (green-colored line: pH 7, 10^7^ PFU/mL; red-colored line: pH 6, 0.9 × 10^7^ PFU/mL) The dataset of HCoV-229E in neutral medium in this plot is the same as (**C**) for the comparison.

Theoretically, for the spherical-shaped corona virus, the first two absorption frequencies correspond to the structural resonant dipolar modes of spherical harmonics are determined by its elasticity and radius. To measure the frequencies, we firstly designed a sensing device for virions using coplanar waveguide (CPW) coated with a nano-thickness hydrophobic self-assembled monolayer mask (See Fig. 1 and “Methods” section). The dipolar mode frequencies and absorption efficiency of SARS-CoV-2 were then successfully characterized by calculating the normalized insertion loss following the measurement via a microwave vector network analyzer. The SARS-CoV-2 virions were first isolated, stored and diluted in Dulbecco's Modified Eagle Medium (pH 7 ~ 7.4) at a series of virus titer of 10^7^, 10^6^, and 10^5^ PFU/mL. The schematic illustration of the viral resonant modes and normalized microwave IL spectra results for different virus titer are shown in Fig. 2A and B, respectively.

In Fig. 2B, the first absorption peak can be observed at 4 GHz with a normalized IL value over 32% and a full width at half maximum (FWHM) bandwidth of approximately 1 GHz for a viral concentration of 10^7^ PFU/mL. The uncertainty of our measured frequency of the absorption peak is negligible (See Quantification and Analysis in Method section). Therefore, all spectra results in Fig. 2B–D were calculated from a single measurement for the control and experimental groups. The diameters of the SARS-CoV-2 virions range from 90 to 110 nm^15^ and resonant frequency is inversely proportional to the diameter of virions, which results in a rather large bandwidth. For lower virus titer, although the resonant frequency peak and the bandwidth were independent to the titer, the normalized IL value was nonlinear to the viral concentration. For a virus titer of 10^5^ PFU/mL comparing to 10^7^ PFU/mL, the absorption peak value only decreased to 25% (absorption decreases from 32 to 8%). This phenomenon can be attributed to the non-homogeneous spatial distribution of virions in the medium above the detection area of the CPW. The interaction of the microwaves with the adjacent environment depends on the extended out electric field; therefore, the sensitivity is higher for virions closer to the gap beside the signal line. This results in a still strong signal from the virions that are concentrated at the bottom of the medium.

We also observed a second MRA peak at 7.5 GHz, which is almost two times higher than the first resonant frequency with roughly 1.5 to 2 GHz bandwidth. In our previous study, we have explained the mechanism wherein the electric field induced core–shell displacement results in structural resonance of dipole modes of spherical (SPH) harmonics^4–6^. Therefore, the first MRA peak at 4 GHz represents the fundamental dipolar mode [SPH, *l* = 1, n = 0] (Fig. 2A, top) and the second MRA peak at 7.5 GHz represents the higher dipolar mode [SPH, *l* = 1, n = 1] (Fig. 2A, bottom). Here *l* and n denote the angular order number and overtone number respectively. According to Lamb’s theory, only the spherical modes with *l* = 1 can induce a dipolar coupling with the electromagnetic waves. The selection rule tells us that the vibration modes are infrared active only when the dipole moment changes during the vibrations process. Therefore, the fundamental breathing mode (*l* = 0) cannot be induced by microwave, and the corresponding absorption peaks could not be measured^16^.

### Dipolar modes frequencies of SARS-CoV-2 compared with HCoV-229E

We assessed the spectra of HCoV-229E virions for the same medium conditions as SARS-CoV-2, and both media are neutral with pH values of 7–7.4 (Fig. 2C). For the 10^7^ PFU/mL virus titer, spectrum for HCoV-229E (Green line in Fig. 2C) also exhibited two dipolar-mode-related peaks in the figure. The first dipolar mode resonant frequency shifted to a higher frequency of 4.2 GHz than that of SARS-CoV-2 with a normalized IL value of around 19%; the second resonant frequency remained at 7.5 GHz. This result confirms that the mass, size, and composition of the virions are slightly different for both viruses, even though they belong to the same subfamily under the Nidovirales order.

### Dipolar modes frequencies of HCoV-229E in weak acidity medium

We then assessed the spectra of HCoV-229E virions in weak acid medium with the virus titer close to the previous neutral sample (Fig. 2D). It is noted that the normal pH range for saliva is 6.2 to 7.6 with an average value of 6.7^17^. Widely considering the respiratory mucosa, such as nasal, tracheal, or bronchial, the secretion is reported to be weak acid with pH values of 6–7^18^. In order to further investigate the effects of the acidity levels on the SRET-induced resonant frequency, we changed the pH level by adding KH_2_PO_4_ solution to the viral solution without causing noticeable change to the original virus titer (10^7^ PFU/mL). The newly obtained viral solution to be compared with the neutral one had a pH level and virus titer of 6 and 0.9 × 10^7^ PFU/mL, respectively. The normalized IL spectrum for the sample was shown in Fig. 2D. The 1st order dipole mode remains unchanged except the absorption efficiency decreased from 19 to 5% under similar virus titer. By comparing the frequency of 2nd order dipole resonant mode, a shift was observed from 7.6 to 7.8 GHz. This variation of the absorption efficiency can be explained by the zeta potential originating from the surface charges of the virions and the ions in the medium. Typically, decreasing pH level by adding acid will increase the zeta potential^19^, thereby changing the tendency of the virions to flocculate. The unchanged frequency of 1st dipolar mode indicated that the virions are still completely dispersed with a thick hydration layer or an electric double layer on the surface. The virions had higher zeta potential at lower pH level caused by the adsorption of more positive ions on the virion surfaces. This further reduces the effective dipole charges of the core–shell separation structure because of the screening effect from the additional charges. This decrease in the dipole charge would in-turn decrease the microwave absorption cross-sections. The SRET process mostly depends on the dipole charge and the strength of the applied electric field; therefore, the normalized IL values decreased in weakly acidic medium.

### Quality factor of the spectra

We defined the quality factor of the MRA (QMRA) as the ratio of the peak frequency to the bandwidth (here defined as the FWHM of the absorption peak). For first dipolar mode (*l* = 1, n = 0) of SARS-CoV-2 with the highest concentration (10^7^ PFU/mL), the QMRA was calculated to be 4.2. As the viral concentration was decreased to 10^6^ and 10^5^ PFU/mL, the QMRA values increased to 4.7 and 5, respectively. For HCoV-229E, the QMRA value was 4.9 for the first dipolar mode for the same viral concentration (10^7^ PFU/mL) in the same medium as that used for SARS-CoV-2; this QMRA value decreased to 4.4 in both weakly acidic and PBS mediums (See Supplementary Fig. 3). Our previous study on the Influenza A and the Perina nuda viruses (PnV)^5^ discussed the QMRA broadening effect caused by size distribution and the viscous damping effect of the water. The size distribution research on SARS-CoV and SARS-CoV-2 using transmission electron microscopy^15^ has determined the average diameter of SARS-CoV-2 to be 100 nm with a ± 10% size variation. The corresponding quality factor would be approximately 5, which corroborates our results with all the reported QMRA values of lower than 5. Based on the theoretical simulation on polymethylmethacrylate (PMMA) nanoparticles to imitate the mechanical properties of virions, viscous water could strongly damp the vibrations and decrease the quality factor of the first dipole mode down to 3 ~ 5. Both the aforementioned factors contribute almost equally to the broadening of the absorption peak, resulting in a QMRA of between 4 and 5.

## Conclusion

Overall comparisons of the normalized microwave IL spectra of influenza A and SARS-CoV-2 revealed that the first dipolar mode frequency decreased from 8.2 to 4 GHz, while the normalized IL value increased from 23 to 32%. For safe sterilization through virion envelope rupture, lower operating frequency results in lower loss in aerosol considering the dielectric loss of water. Moreover, higher absorption efficiency suggests that SARS-CoV-2 inhibition might be achieved at a microwave power density that is safe for a human body.

In summary, by measuring the normalized insertion loss spectra, the microwave resonant frequencies of SARS-CoV-2 and HCoV-229E were successfully identified. First, for the lowest viral concentration, the minimum number of SARS-CoV-2 infectious virions was determined to be 4000, and significant MRA peaks can be identified with our system. Second, the virus titer, medium, and the acidity level did not change the resonant frequencies. Finally, identification of this resonant frequencies of 4 GHz and 7.5 GHz and the relatively high microwave absorption efficiency increase our confidence to the potential realization for a SARS-CoV-2 sterilization and COVID-19 transmission control using high penetration microwaves in free space with a power level safe to the open public.

## Methods

### Viral production of SARS-CoV-2 and HCoV-229E

The cell line and SARS-CoV-2 virus were prepared in a biosafety level-3 (BSL-3) laboratory in National Taiwan University Hospital. For the cell line, Vero E6 cells were purchased from American Type Culture Collection (ATCC) (Manassas, VA, USA) and were grown in Dulbecco’s modified Eagle’s medium (DMEM) containing 10% fetal bovine serum (FBS) (Life Technologies), 1% antibiotic. The cells were cultured at 37 °C with 5% CO_2_. The SARS-CoV-2/NTU03/TWN/human/2020 (Accession ID EPI_ISL_413592) viruses were isolated from SARS-CoV-2-infected patient throat swab and propagated in Vero E6 cells in DMEM supplemented with 2 μg/ml tosylsulfonyl phenylalantyl chloromethylketone (TPCK)-trypsin (Sigma-Aldrich), and stored at − 80 °C until further study. The virus titers were determined by plaque assay for subsequent analysis. The experiments were performed in accordance with relevant local guidelines and regulations. The experimental protocols were approved by the NTUH Research Ethics Committee (202002002RIND by Dr. Jann-Tay Wang), and the informed consent was obtained from all participants. For HCoV-229E, the cell line and the virus were prepared in a BSL-2 laboratory in Chang Gung University, Taiwan. For the cell line, HuH7 (American Type Culture Collection, Manassas, VA, USA) cells were grown in Dulbecco’s modified Eagle’s medium (DMEM) containing 10% fetal bovine serum (FBS), 1% antibiotic/antimycotic solution, and 1% l-glutamine (Gibco, Grand Island, NY, USA). The cells were cultured at 37 °C with 5% CO_2_. The HCoV-229E viruses were isolated from Chang Gung Memorial Hospital and propagated in HuH7 cells, maintained in DMEM with 2% FBS, and stored at − 80 °C until further study.

### Acidic medium preparation

We maintained the virus titer in DMEM of close to 10^7^ PFU/mL and changed the pH level by adding a small amount of 1% KH_2_PO_4_ solution to the viral solution. The 1% KH_2_PO_4_ solution was first prepared by mixing 1 g of KH_2_PO_4_ powder into 100 mL deionized water with a final pH level of 4.52, which was measured using a pH meter. After a 100 × dilution of the 1% KH_2_PO_4_, the pH level reached 5.17. Finally, 20 µL of the KH_2_PO_4_ solution with a pH of 5.17 was added to 180 µL of the viral solution with a pH of 7 and a concentration of 10^7^ PFU/mL. The newly obtained viral solution had a pH level and concentration of 6 and 0.9 × 10^7^ PFU/mL, respectively. All the processes were conducted in a BSL-2 laboratory in Chang Gung University, Taiwan.

### CPW with SAM mask

In order to realize the resonant frequency which high energy transfer took place, we need to scan the reasonable microwave frequency range to find the energy loss dip on spectrum. From our previous work, by considering the resonance process that core–shell is oscillating in the opposite directions, we can estimate that the resonant frequency should be lower than 8 GHz since the reduced mass^20^ from two-body problem is larger than influenza A virus. We can predict that if there exists the higher order resonant mode, then we should be able to find it within 16 GHz. Therefore, we first design a transmission line using CPW as a two-port network of which the microwave EM signal is able to interact with the environment (i.e. the medium containing SARS-CoV-2), and the network properties like transmittance, reflectance, and insertion loss (IL) can be measured by a two-port vector network analyzer.

CPW is a well-known basic waveguide for microwave application that features the coplanar signal line and two ground planes, which are symmetrically synthesized on top of substrate. The purpose of the SAM mask is to enhance the repeatability of the coupled microwave transmission to the medium containing SARS-CoV-2 particles by fixing the interaction area. To make a CPW, we choose a PCB board with 100 um copper film covered on top as a substrate. The permittivity ${\varepsilon }_{PCB}$ of the PCB is 4.2. We etched the copper film to form the two gaps between the signal line and two grounds with the aid of photoresist. See Fig. 1A, the width of the signal line and the gap is 2.1 mm and 0.55 mm respectively so that the input impedance from both end is 50 Ohm. The length between both ends of the CPW is 2.5 cm and two SMA connectors were soldered on both ends.

Since the samples under test in this study is cultivated and purified in Dulbecco's modified Eagle’s medium (DMEM) and phosphate buffered saline (PBS) medium, which are both aqueous liquid, the contact area on the CPW is hard to keep identical for experimental and control group. In addition, the change of the contact area will also result to the change of the S-parameters. Therefore, we apply a simple but useful method to confine the liquid sample in order to keep the detection volume identical for every measurement. We use spray coating method to apply a self-assembled hydrophobic nanoscale monolayer using MSG-022 with contact angle over 110° (purchased from Giant Nano Technology Co., Ltd.). The self-assembled monolayer (SAM) is designed to be hollow in the center so that the strip line and the gaps are both exposed to the environment (Fig. 1B). We first use a rubber cubic with flat and square shape to cover the detection area with CPW beneath. SAM is synthesized on the area outside of the rubber cubic in order to create a square area (5 mm × 5 mm) with less hydrophobicity. Any other kind of SAM materials with fluorocarbon chain or hydrocarbon chain such as octadecyltrichlorosilane will also work. The aqueous liquid dipped inside the square area will be confined and the boundary of the aqueous liquid is thus well defined by the SAM (Fig. 1E). The SAM film thickness is less than 10 nm with dielectric constant lower than 1.4 and the film is also non-conductive. The SAM with air-like dielectric constant brings negligible change to the impedance of CPW so that the properties of 2-port network remain nearly the same after SAM treatment. The one-step synthetization of nano-thickness SAM by spraying or spin-coating makes it easy to produce. Since the detection process requires a control group, the well-defined area of the aqueous liquid brings the reliable repeatability of the measured spectrum.

### S-parameters scanning using vector network analyzer

After the CPW detection device was fabricated, we used MS2028C, Anritsu portable VNA with frequency bandwidth 5 kHz–20 GHz and dynamics range over 85 dB to measure the 2-port S-parameters of CPW with SAM mask to ensure that the S_21_ parameter is close to 0 dB for good power feed-in. Later, the CPW device and the VNA were moved into BSL-2 and BSL-3 labs and calibrated using the 50 Ohm VNA calibration kits for the following experiments of HCoV-229E and SARS-CoV-2 respectively. For a 2-port network device, the S-parameters matrix can be used to describe the transmittance (S_12_ and S_21_) and reflectance (S_11_ and S_22_). Apart from that, the energy of signal has either a gain or a loss inside the device. For a transmission line, the energy of EM wave is gradually lost when propagating, and it is usually referred as insertion loss (IL). The relation of S-parameters and IL can be written as ${\left|{S}_{11}\right|}^{2}+{\left|{S}_{21}\right|}^{2}+IL=1$. When a medium is in contact with the CPW, the medium caused a permittivity change to the transmission line and not only the S-parameters are affected, but the IL is also increased if there exists an energy transfer mechanism in the medium. With a fixed volume of the medium on CPW and well-defined interaction area, the small amount of the suspended particles would not result in an obvious change to the IL. However, if the particles provide an energy transfer pathway, then the IL would be affected. To determine the bandwidth of our results, as we can see in the Supplementary Fig. 1, an obvious difference can be observed in the microwave attenuation spectra within 10 GHz by comparing both cases, which are with virus (green thin line) and without virus (red thin line). Since the medium itself would contribute rather high insertion loss (> 90%) to the CPW transmission line at high frequency (10 GHz to 20 GHz), the noise would be amplified when calculating the normalized IL. Therefore, we only consider the bandwidth from 0.2 GHz to 10 GHz which has better SNR. In the measurement of SARS-CoV-2, due to the difficulty of achieving perfect calibration of VNA in BSL-3 lab, the sum of ${\left|{S}_{11}\right|}^{2}$ and ${\left|{S}_{21}\right|}^{2}$ is slightly over 1 which lead to a gain in this 2-port system. Therefore, we had to omit the components which its frequency is lower than 1.1 GHz.

### Experimental flow of measuring the S-parameters in a biosafety cabinet

Firstly, the vector network analyzer was connected with two high frequency bandwidth (DC ~ 18 GHz) signal lines with SMA connectors and calibrated by calibration kit. After calibration, the CPW sensing device was mounted using a PCB fixture in biosafety cabinet and connected with the signal lines (Fig. 1D). After setting up the system, we prepared two samples which are the medium and the same medium with virions. For each formal measurement, the total sample volume dripped on the square detection area was fixed at 40 µL, ensuring that at least 4000 infectious virions were present in the lowest virus titer sample (10^5^ PFU/mL). In the acquisition process of the normalized IL spectra of the coronavirus, we need to go through two separate measurements on the same detection CPW device. The first one, which serves as the control group, is the fixed amount of medium without any coronavirus. The second one, which serves as the experimental group, is the same amount of identical medium as control group but with coronavirus purified and suspended inside. First, use a pipet to drop the 40 µL medium on the sensing square area and measure the full 2-port S-parameters. After the data was acquired, remove the medium using the pipet and make sure the sensing area is clean and dry. Finally, do the same measurement using the medium with virions. These two separate data can be used to calculate the normalized insertion loss spectrum afterward. All the processes were conducted in a BSL-3 lab and a BSL-2 lab for the S-parameters measurements of SARS-CoV-2 and HCoV-229E respectively.

### Quantification and analysis

If the full 2-port S-parameters of two groups can be measured, with system setup and samples geometry kept identical for two measurements, the microwave attenuation spectra $A\left(f\right)$ can be calculated as ${\left|{S}_{11}\right|}^{2}+{\left|{S}_{21}\right|}^{2}=1-IL$. Finally, the normalized IL of the coronavirus can be calculated by the following relation, which “v + b” and “b” represent the “virus + medium background” and “medium background”:

$$\text{Normalized} \, \text{IL} = 1-\frac{{A}_{v+b}\left(f\right)}{{A}_{b}\left(f\right)}$$

For the control and experimental groups, two spectra of S-parameters have been measured individually, and thus we can acquire 4 normalized IL spectra by cross comparison. We statistically calculate the standard error of the mean of the resonant central frequencies and the uncertainty from our results are tens of MHz. Since the slight variation from our measurements can be negligible comparing to the gigahertz dipolar mode frequency and bandwidth, we showed the normalized IL by a single measurement result. Our measured S-parameters and normalized IL spectra have a 0.05 GHz frequency resolution and a Savitzky–Golay filter with the window size of 20 data points was applied to smooth the lines in graph.

## Supplementary Information

Supplementary Information.

## Data Availability

All original code and the raw data for calculation of the figures in the article have been deposited at google cloud space and is publicly available as of the date of publication. (https://drive.google.com/drive/folders/1BWKOz_TvoZngHrRvkA49JSUsZ1FGoUIZ?usp=sharing).
