# Supplementary material for: Microwave resonant absorption of SARS-CoV-2 viruses
Source: Sci Rep. 2022 Jul 22;12:12596. doi: 10.1038/s41598-022-16845-5 (PMC9307221; doi:10.1038/s41598-022-16845-5)
Supplement: Supplementary file 1 — Supplementary Information. [file 41598_2022_16845_MOESM1_ESM.docx]

Supplementary Information

Microwave Resonant Absorption of SARS-CoV-2 Viruses

**Verification of the efficient detection area on CPW**

Here we also showed the calculated normalized IL spectrum if we placed 10uL of the same HCoV-229E sample only on the signal line without covering the gap (conducted in a BSL-2 lab in Chang Gung University, Taiwan). In this case, the normalized IL spectrum is flattened and close to zero **(See supplementary Fig. 2)**, which means that the electric field is mainly confined in the space inside the gap between the signal and the ground. Therefore, the virions particles are not able to interact with the transmitted microwave and cause significant power loss.

**Normalized insertion loss spectra of HCoV-229E in different medium**

To understand the effect of medium type on the SRET-induced resonant frequency, we purified the HCoV-229E virions in PBS (pH = 7~7.4) with a virus titer of 10^5^ PFU/mL (conducted in a BSL-2 lab in Chang Gung University, Taiwan); the normalized IL spectrum is represented by the red-colored line in **supplementary figure 3**. The resonant frequencies, bandwidths, and other profile features remained the same with only the normalized IL value reducing from 19% to 6 % (3.1 times). Compared to previous acidic conditions, its normalized IL value (5 %) at the resonant frequency is almost the same as that (6%) for the lowest concentration of virus in PBS, even though the virus titer is approximately 90 times higher. In addition to confirming that the virus titer and ionic composition only affect the total microwave absorption, this result also implies that the medium composition under same acidity level would not result in a frequency shift.

**Supplementary Figures**


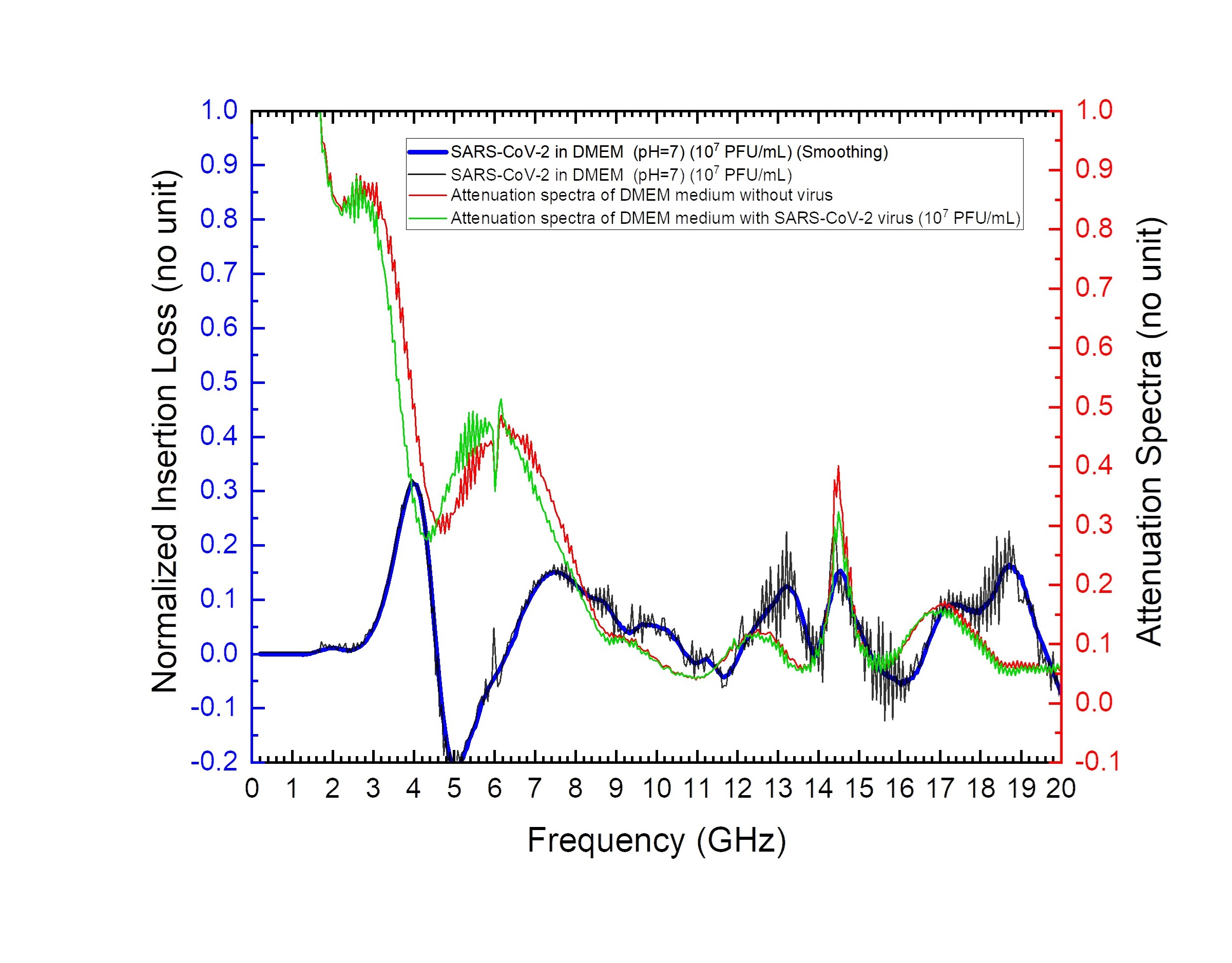


**Supplementary figure 1.** Summation of transmission and reflection intensity of both control and experimental groups, and the calculated normalized insertion loss spectra of SARS-CoV-2.


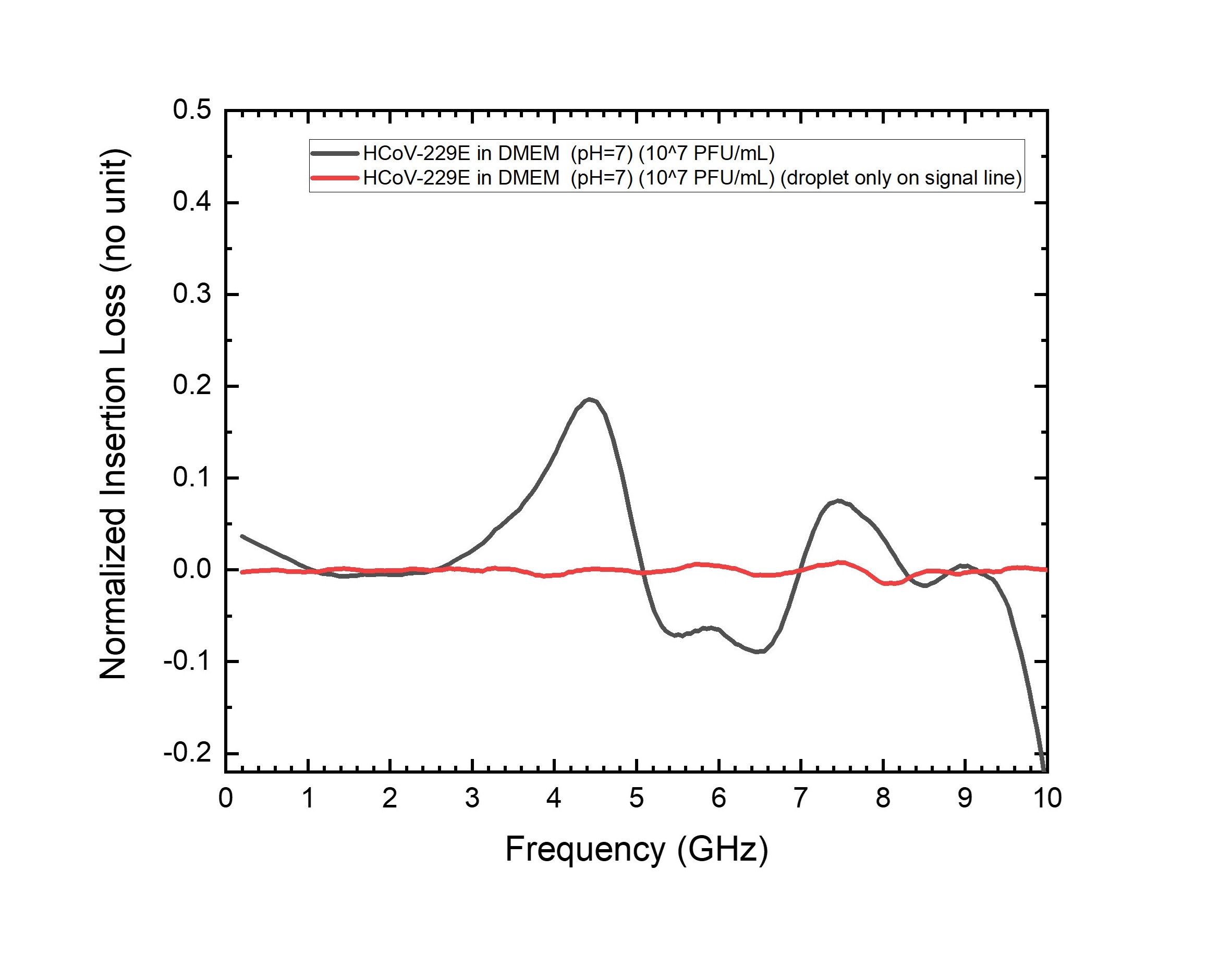


**Supplementary figure 2.** Normalized insertion loss spectrum of the HCoV-229E compared with the condition that the droplet sample is placed on top of the signal line without crossing the gap of CPW.


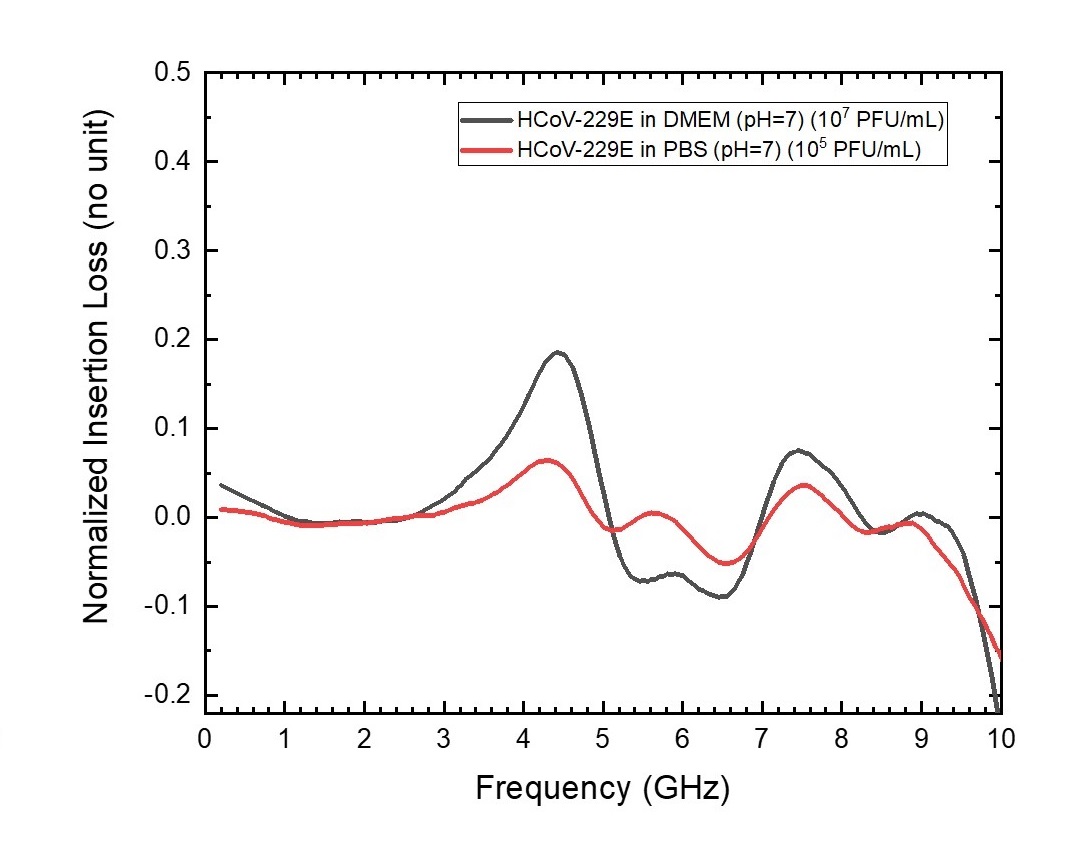


**Supplementary figure 3.** Normalized insertion loss of HCoV-229E purified in different media, DMEM and PBS, with the same acidity levels. (Black-colored line: DMEM, 10^7^ PFU/mL; red-colored line: PBS, 10^5^ PFU/mL)
